# High-Sensitivity Troponin and the Application of Risk Stratification Thresholds in Patients With Suspected Acute Coronary Syndrome

**DOI:** 10.1161/CIRCULATIONAHA.119.042866

**Published:** 2019-09-01

**Authors:** Anda Bularga, Kuan Ken Lee, Stacey Stewart, Amy V. Ferry, Andrew R. Chapman, Lucy Marshall, Fiona E. Strachan, Anne Cruickshank, Donogh Maguire, Colin Berry, Iain Findlay, Anoop S.V. Shah, David E. Newby, Nicholas L. Mills, Atul Anand

**Affiliations:** 1 British Heart Foundation Centre for Cardiovascular Science (A.B., K.K.L., S.S., A.V.F., A.R.C., L.M., F.E.S., D.E.N., A.S.V.S., N.L.M., A.A.), University of Edinburgh, United Kingdom.; 2 Usher Institute of Population Health Sciences and Informatics (A.S.V.S., N.L.M.), University of Edinburgh, United Kingdom.; 3 Department of Biochemistry, Queen Elizabeth University Hospital, Glasgow, United Kingdom (A.C.).; 4 Emergency Medicine Department, Glasgow Royal Infirmary, United Kingdom (D.M.).; 5 Institute of Cardiovascular and Medical Sciences, University of Glasgow, United Kingdom (C.B.).; 6 Department of Cardiology, Royal Alexandra Hospital, Paisley, United Kingdom (I.F.).

**Keywords:** acute coronary syndrome, myocardial infarction, risk stratification, troponin

## Abstract

Guidelines acknowledge the emerging role of high-sensitivity cardiac troponin (hs-cTnl) for risk stratification and the early rule-out of myocardial infarction, but multiple thresholds have been described. We evaluate the safety and effectiveness of risk stratification thresholds in patients with suspected acute coronary syndrome.

**Methods:**

Consecutive patients with suspected acute coronary syndrome (n=48 282) were enrolled in a multicenter trial across 10 hospitals in Scotland. In a prespecified secondary and observational analysis, we compared the performance of the limit of detection (<2 ng/L) and an optimized risk stratification threshold (<5 ng/L) using the Abbott high-sensitivity troponin I assay. Patients with myocardial injury at presentation, with ≤2 hours of symptoms or with ST-segment elevation myocardial infarction were excluded. The negative predictive value was determined in all patients and in subgroups for a primary outcome of myocardial infarction or cardiac death within 30 days. The secondary outcome was myocardial infarction or cardiac death at 12 months, with risk modeled using logistic regression adjusted for age and sex.

**Results:**

In total, 32 837 consecutive patients (61±17 years, 47% female) were included, of whom 23 260 (71%) and 12,716 (39%) had hs-cTnl concentrations of <5 ng/L and <2 ng/L at presentation. The negative predictive value for the primary outcome was 99.8% (95% CI, 99.7%–99.8%) and 99.9% (95% CI, 99.8%–99.9%) in those with hs-cTnl concentrations of <5 ng/L and <2 ng/L, respectively. At both thresholds, the negative predictive value was consistent in men and women and across all age groups, although the proportion of patients identified as low risk fell with increasing age. Compared with patients with hs-cTnl concentrations of ≥5 ng/L but <99th centile, the risk of myocardial infarction or cardiac death at 12 months was 77% lower in those <5 ng/L (5.3% vs 0.7%; adjusted odds ratio, 0.23 [95% CI, 0.19–0.28]) and 80% lower in those <2 ng/L (5.3% vs 0.3%; adjusted odds ratio, 0.20 [95% CI, 0.14–0.29]).

**Conclusions:**

Use of risk stratification thresholds for hs-cTnl identify patients with suspected acute coronary syndrome and at least 2 hours of symptoms as low risk at presentation irrespective of age and sex.

**Clinical Trial Registration:**

URL: https://www.clinicaltrials.gov. Unique identifier: NCT01852123.

## Abstract 2

Supplemental Digital Content is available in the text.

**Clinical Perspective**

**What Is New?**

- In 32 837 consecutive patients with suspected acute coronary syndrome and at least 2 hours of symptoms, we evaluated the performance of 2 risk stratification thresholds for a high-sensitivity cardiac troponin I assay.
- An optimized risk stratification threshold of <5 ng/L identified twice as many patients at presentation as low risk compared with the limit of detection (<2 ng/L), with an equivalent negative predictive value for myocardial infarction or cardiac death at 30 days.
- Compared with the diagnostic threshold, patients with cardiac troponin I concentrations <2 ng/L or <5 ng/L were 80% and 77% lower risk of subsequent cardiac events at 12 months, respectively.

**What Are the Clinical Implications?**

- The use of separate risk stratification and diagnostic thresholds for high-sensitivity cardiac troponin will improve the safety of our assessment of cardiovascular risk in patients with suspected acute coronary syndrome.
- Incorporating a risk stratification threshold into the early evaluation of these patients will enable the majority of patients to avoid unnecessary hospital admission with major benefits for patients and healthcare providers.

The way in which cardiac troponin testing is used in clinical practice is evolving rapidly in parallel with major improvements in assay precision and sensitivity.^1,2^ High-sensitivity cardiac troponin assays are essential for the diagnosis of acute myocardial infarction but are increasingly also used in the assessment of cardiovascular risk to identify patients in the emergency department who are low risk and could be directly discharged.^3–9^ Given that fewer than 10% of patients with suspected acute coronary syndrome have myocardial infarction,^10^ this application of high-sensitivity cardiac troponin testing has major potential to reduce unnecessary hospital admissions with benefits for patients and healthcare providers.

Although the universal definition of myocardial infarction recommends the use of sex-specific 99th centile or upper reference limits from a normal reference population as the diagnostic threshold for myocardial infarction,^3^ there is less consensus on the optimal troponin threshold for the evaluation of cardiovascular risk.^4,5^ The ideal risk stratification threshold would permit the greatest number of patients without myocardial infarction to be classified as low risk without compromising safety. The limit of detection has been proposed,^11–13^ but assay performance at this level is variable, potentially reducing the consistency and effectiveness of this approach.^14–17^ We previously defined the optimal risk stratification threshold as the highest troponin concentration that gave a negative predictive value for myocardial infarction or cardiac death at 30 days of at least 99.5%^6^ to maximize the number of patients identified as low risk while maintaining safety. This was achieved using a high-sensitivity cardiac troponin I assay at a concentration <5 ng/L, which identified two-thirds of patients as low risk at presentation and misclassified fewer than 1 in 200 patients. The only subgroup that did not meet this target for safety were those who presented within 2 hours of symptoms onset, and guidelines now clearly state that serial testing is required in these early presenters.^3,7^

The use of risk stratification thresholds in diagnostic pathways has been evaluated in retrospective analyses of cohort studies^8,9^ but have not been prospectively validated.^4,18^ Many approaches have been proposed, often in small cohorts of selected patients attending a single center, with a limited number of patients in high-risk subgroups. As such, there remains uncertainty as to the performance of these thresholds in practice, where patients are often older and more likely to have comorbidities. Our aim was to compare the diagnostic performance of an optimized risk stratification threshold with the limit of detection, in the patient population in whom risk stratification thresholds have been advocated by international guidelines.^3^ In a prespecified secondary and observational analysis of a multicenter trial of consecutive patients with suspected acute coronary syndrome, we evaluate diagnostic performance in patients presenting with at least 2 hours of symptoms by age and in subgroups to provide reliable estimates for clinical practice. In a substudy of the trial population, we explore the generalizability of this approach by evaluating performance of these risk stratification thresholds across different high-sensitivity assays.

## Methods

### Transparency and Openness Promotion

The trial makes use of multiple routine electronic health care data sources that are linked, deidentified, and held in our national safe haven, which is accessible by approved individuals who have undertaken the necessary governance training. Summary data and the analysis code can be made available upon request from the corresponding author.

### Study Population

High-STEACS (High-Sensitivity Troponin in the Evaluation of Patients With Suspected Acute Coronary Syndrome) was a stepped-wedge cluster randomized controlled trial that evaluated the implementation of a high-sensitivity cardiac troponin I assay in consecutive patients presenting with suspected acute coronary syndrome across 10 secondary and tertiary hospitals in Scotland (URL: https://www.clinicaltrials.gov. Unique identifier: NCT01852123). The study design has been described in detail previously^19^ and was conducted with the approval of the Scotland Research Ethics Committee in accordance with the Declaration of Helsinki. Individual patient consent was not sought. This approach ensured that consecutive patients presenting with suspected acute coronary syndrome were included without selection bias. All patients presenting to emergency departments between June 10, 2013, and March 3, 2016, were screened by the attending clinician and prospectively included in the trial if cardiac troponin was requested for suspected acute coronary syndrome.

For this prespecified secondary and observational analysis, we evaluate the performance of high-sensitivity cardiac troponin I in patients without evidence of myocardial injury at presentation (cardiac troponin concentrations below the sex-specific 99th centile), excluding those patients who presented early (≤2 hours from symptom onset to the initial blood draw), or those with a ST-segment elevation myocardial infarction.

### Substudy Population

To evaluate the generalizability of risk stratification thresholds we used stored samples from a substudy of the trial to compare the performance of different high-sensitivity cardiac troponin I assays (Abbott ARCHITECT_*STAT*_ and Siemens Atellica, Siemens Healthineers) and high-sensitivity cardiac troponin T (Roche Elycsys, Roche Diagnostics). Participants provided informed consent for additional blood sampling and storage, as described previously.^20–22^ The analysis population was defined in the substudy using the same inclusion and exclusion criteria as for the trial population.

### Cardiac Troponin Testing

As previously described, cardiac troponin testing was performed at presentation and repeated 6 or 12 hours after the onset of symptoms at the discretion of the attending clinician in accordance with national and international guidelines in use during enrollment.^19,23,24^ In all patients during both phases of the trial, cardiac troponin was measured using the ARCHITECT_*STAT*_ high-sensitive troponin I assay (Abbott Laboratories, Abbott Park, IL). This assay has a limit of detection of between 1.2 ng/L and 1.9 ng/L,^25^ and for consistency with prior studies we defined this as any concentration <2 ng/L.^26^ For the purpose of this analysis, all patients with an undetectable troponin concentration were assigned a value of 1.0 ng/L. The inter-assay coefficient of variation is less than 10% at 4.7 ng/L and the sex-specific 99th centile diagnostic thresholds are 16 ng/L for women and 34 ng/L for men.^27^ High-sensitivity cardiac troponin I concentrations were only disclosed to clinicians during the implementation phase of the trial, but given risk stratification thresholds were not used to guide clinical decisions we pooled data from both phases of the trial for the purpose of this analysis.

In the substudy, samples were also analyzed using the Siemens Atellica high-sensitivity cardiac troponin I assay and Roche Elycsys high-sensitivity cardiac troponin T assays.^5,22^ For these assays the limit of detection is 1.6 ng/L and 5 ng/L respectively, and the limit of blank for the cardiac troponin T assay is 3 ng/L. For all 3 assays, we evaluated performance of the risk stratification threshold of 5 ng/L, the lower threshold of <2 ng/L for cardiac troponin I, and <3 ng/L for cardiac troponin T, as these thresholds are equivalent to the limit of detection and limit of blank, respectively.

### Adjudication of the Diagnosis of Myocardial Infarction

Clinical information was collected from a standardized electronic patient record (TrakCare; InterSystems Corporation, Cambridge, MA) linked to local and national datasets. Electrocardiographic data including algorithmic interpretation was available by electronic capture in a subgroup of patients (MUSE, GE Healthcare). All unique interpretation codes generated by this system (n=4291) were reviewed by a consensus panel who selected codes consistent with possible ischemia (n=180). Example electrocardiograms featuring these codes were then reviewed independently by at least 2 physicians to determine reliability for clinically significant myocardial ischemia. The final list of 119 codes (see Appendix in the online-only Data Supplement) were then applied to the study population with electronic electrocardiograms to determine whether myocardial ischemia was present for each patient.

Two physicians from our adjudication panel independently reviewed all clinical information to classify patients with any high-sensitivity cardiac troponin measurement >99th centile on serial testing during the index presentation in accordance with the third Universal Definition of Myocardial Infarction.^28^ Myocardial infarction following discharge and all death outcomes were also independently adjudicated by 2 physicians blinded to study phase and any disagreements were resolved by a third physician.

### Study Outcomes

The primary safety outcome was type 1 or 4b myocardial infarction during the index presentation, or subsequent type 1 or 4b myocardial infarction or cardiac death within 30 days of the index presentation. The secondary safety outcome was subsequent type 1 or 4b myocardial infarction or cardiac death at 12 months. Type 2 myocardial infarction was not included in the composite outcome, as by definition these patients present with alternative, often noncardiac conditions that determine whether they require hospital admission or discharge.

The number and proportion of patients with high-sensitivity cardiac troponin concentrations less than 2 ng/L or 5 ng/L at presentation were measured to evaluate effectiveness of these risk stratification thresholds. Secondary outcomes of cardiac catheterization, coronary intervention and new medical therapy were collected from local and national databases as previously described.^19^

### Statistical Analysis

Baseline characteristics are summarized as percentages for categorical variables, mean (standard deviation) or median (interquartile range) as appropriate. The negative predictive value was determined using 2×2 tables to calculate the true and false negative rates for the primary outcome, comparing patients with cardiac troponin concentrations at presentation less than 2 ng/L and less than the risk stratification threshold of 5 ng/L. As we expected the negative predictive value to approach 100%, we estimated the proportion by sampling from a binomial likelihood distribution with a Jeffreys prior, as such approaches have good coverage for proportions that approach 0 or 1 (ß distribution shape parameters both 0.5).^29^ Analysis by stratification was used to compare performance in different subgroups. For age, the negative predictive value was calculated for each integer age value between 20 and 90 years, and plotted with a line of best fit and 95% CI. The negative predictive value was also determined separately in those with and without prior history of ischemic heart disease, diabetes mellitus, stroke, heart failure and renal impairment (estimated glomerular filtration rate <60 mL/min/1.73 m^2^ determined by Modified Diet in Renal Disease equation) or myocardial ischemia on the electrocardiogram at presentation.

For the secondary outcome, the rates of myocardial infarction or cardiac death were compared in patients with cardiac troponin concentration at presentation less than 2 ng/L, less than 5 ng/L, and 5 ng/L to the sex-specific 99th centile. In a post-hoc analysis, we also compared the rates of myocardial infarction or cardiac death in patients with cardiac troponin concentrations between these risk stratification thresholds. Logistic regression modelling for the primary and secondary outcomes was performed using patients with cardiac troponin concentrations between 5 ng/L and the sex-specific 99th centile as a reference group. Odds ratios were adjusted for differences in age and sex. All analyses were performed using R (version 3.5.1).

## Results

The trial enrolled 48 282 consecutive patients (61±17 years, 47% women) across 10 hospitals in Scotland. A total of 32 837 patients (68%) remained in the analysis population (58±1 years, 47% women) after excluding those with cardiac troponin concentrations >99th centile at presentation (n=7795), and those presenting ≤2 hours of symptom onset (n=6469) or with ST-segment elevation myocardial infarction (n=925), and where the high-sensitivity cardiac troponin concentrations at presentation were missing (n=256; Figure I in the online-only Data Supplement).

### Proportion and Characteristics of Patients Identified by Risk Stratification Thresholds

In our analysis population, 23 260 (71%) had a cardiac troponin concentration below 5 ng/L, and 9577 (29%) were between 5 ng/L and the 99th centile. There were 12 716 (39%) patients with cardiac troponin concentrations below 2 ng/L at presentation. Patients with cardiac troponin concentrations below these risk stratification thresholds were younger, more likely to be female, and had fewer cardiovascular risk factors than those with troponin concentrations between 5 ng/L and the 99th centile (Table 1). Similarly, the use of antiplatelet agents and secondary prevention were half as frequent in patients with cardiac troponin concentrations below 5 ng/L compared with those above this threshold. In those below 2 ng/L, even lower rates of cardiovascular risk factors were observed amongst younger, predominantly female patients when compared with those with troponin concentrations 2–4 ng/L (Table I in the online-only Data Supplement).

**Table 1.**
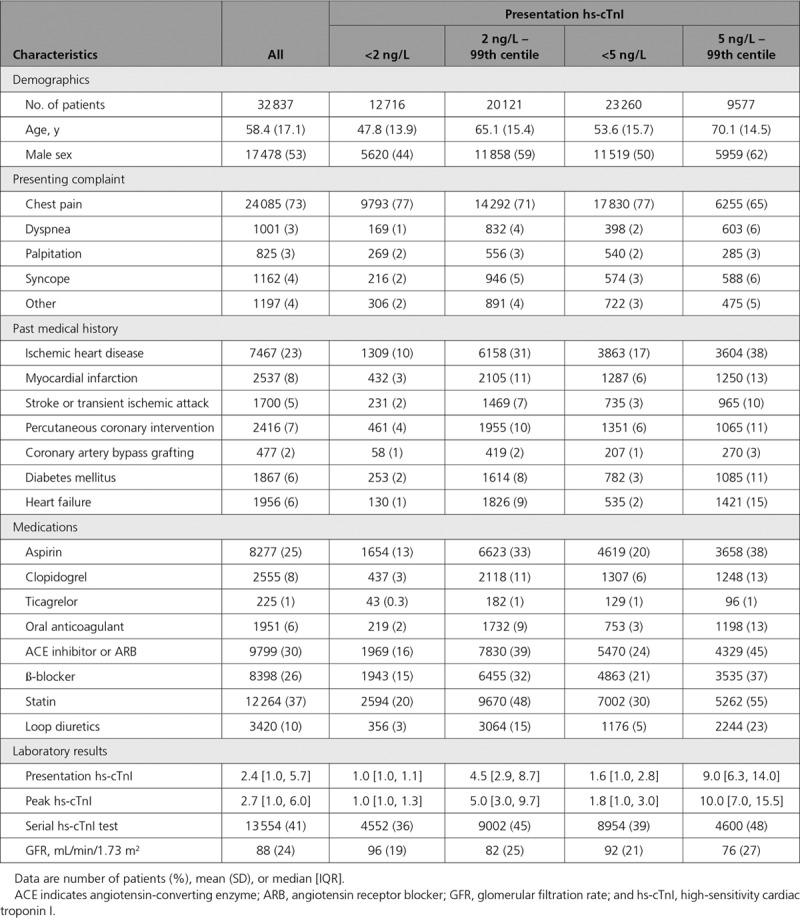
Baseline Characteristics of Participants, by Presentation hs-cTnI

### Diagnostic Performance of Risk Stratification Thresholds

In the analysis population, 1.6% (517 of 32 837) of patients experienced a primary outcome event of index myocardial infarction, or subsequent myocardial infarction or cardiac death within 30 days of presentation. This composite measure included 475 patients with an index myocardial infarction, and 78 and 49 patients with a subsequent myocardial infarction or cardiac death within 30 days, respectively. The majority of composite events occurred in those with cardiac troponin concentrations between 5 ng/L and the 99th centile where the event rate was 4.8% (462 of 9577) at 30 days. There were 55 events in 23 260 patients (0.2%) with cardiac troponin concentrations less than 5 ng/L, and 15 events in the subgroup of 12 716 patients (0.1%) less than 2 ng/L. Of these composite events, cardiac death within 30 days occurred in 45 of 9577 patients with troponin concentrations between 5 ng/L and the 99th centile (0.5%), 4 of 23 260 patients less than 5 ng/L (0.02%) and 1 patient from 12 716 below 2 ng/L (0.01%, Table 2).

**Table 2.**
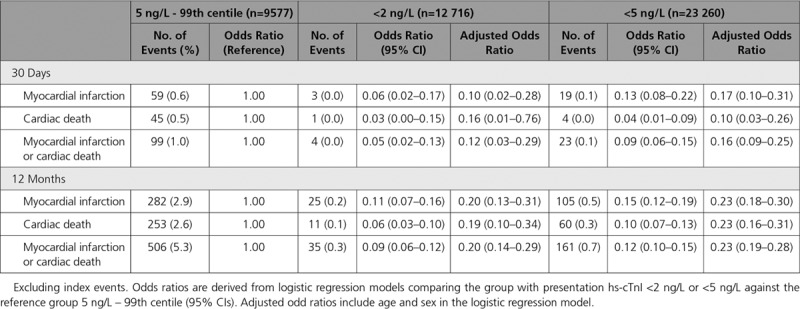
Logistic Regression Modelling for Safety Outcomes at 30 Days and 12 Months, by Presentation hs-cTnI

The negative predictive value for the primary outcome at 30 days in patients with cardiac troponin concentrations less than the risk stratification threshold of 5 ng/L at presentation was 99.8% (95% CI, 99.7%–99.8%). The negative predictive value in the subgroup of patients with cardiac troponin concentrations <2 ng/L was 99.9% (95% CI, 99.8%–99.9%). Although the prevalence of the primary outcome varied between sites (range, 0.8%–2.1%), the negative predictive value remained consistent across all sites (Table II in the online-only Data Supplement). In patients presenting within 2 hours of symptom onset (n=6469), the negative predictive value was lower at both thresholds (99.0% [95% CI, 98.7%–99.3%] for those <5 ng/L and 99.6% [95% CI, 99.3%–99.8%] for patients <2 ng/L, Table III in the online-only Data Supplement). Confusion matrices and other diagnostic metrics for the trial and analysis populations are shown in Tables IV and V in the online-only Data Supplement.

### Diagnostic Performance of Risk Stratification Thresholds in Subgroups

**Figure 1.**
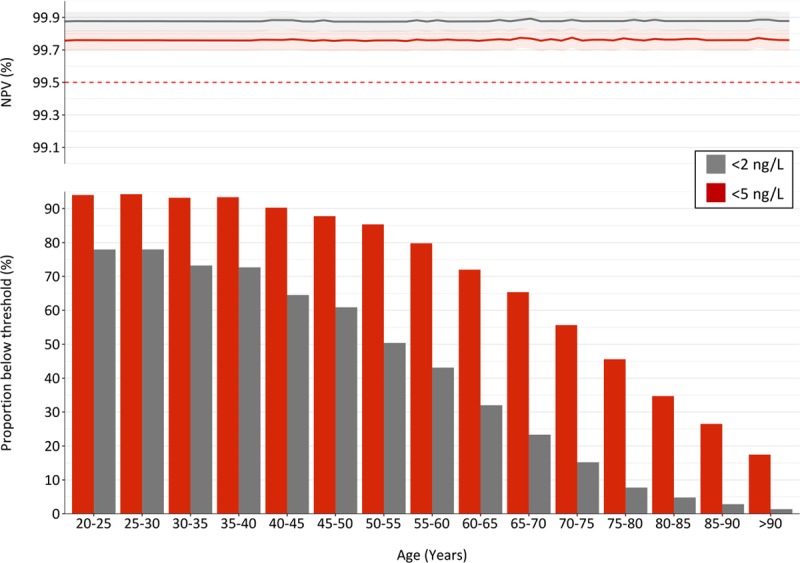
**Performance of cardiac troponin I risk stratification thresholds by age.** Negative predictive value for the primary outcome of myocardial infarction or cardiac death at 30 days across a range of ages with 95% confidence intervals (shaded) for patients with cardiac troponin concentrations below 2 ng/L (gray) and 5 ng/L (red) at presentation. The negative predictive value was calculated for each integer age value between 20 and 90 years, and plotted with a line of best fit and 95% CI. The bar chart shows the proportion of patients in each 5-year age band with cardiac troponin concentrations below each threshold.

The proportion of patients with cardiac troponin concentration below the 5 ng/L and 2 ng/L thresholds varied markedly by age, but the negative predictive value of these approaches to risk stratification were identical across all age groups (Figure 1). The lower bounds of the 95% CI was >99.5% for both thresholds even in the oldest patients. In patients >65 years old (n=11 837), the proportion identified as low risk with a high-sensitivity troponin concentration below the 2 ng/L risk stratification threshold was diminished at only 11% (1303 of 11 837), compared with 46% (5463 of 11 837) with cardiac troponin concentrations <5 ng/L.

**Figure 2.**
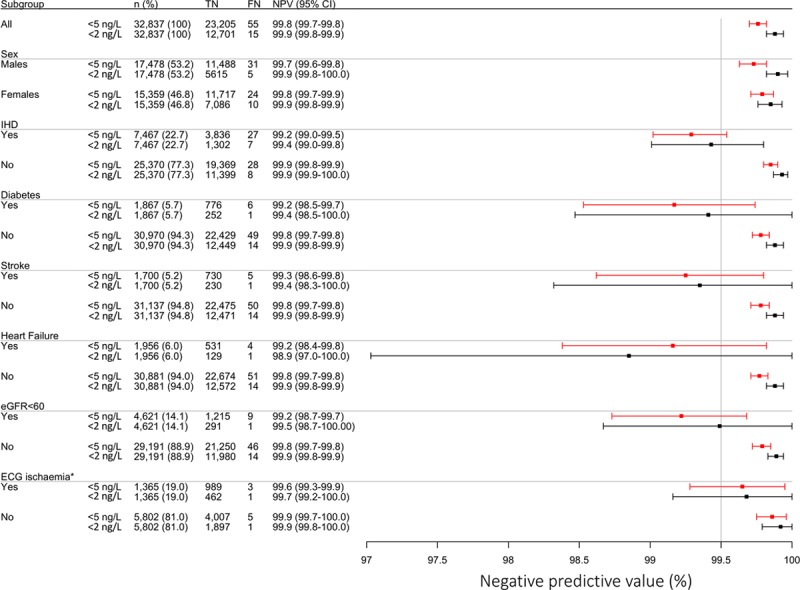
**Safety of cardiac troponin I risk stratification thresholds by subgroups.** Forest plot showing the number of patients in each subgroup, true negatives (TN) and false negatives (FN) with the negative predictive value (NPV) for the primary outcome, stratified by patients with cardiac troponin concentrations below 2 ng/L (black) and below 5 ng/L (red). *ECG ischemia data available in 7167/32 837 (22%) of patients.

Central estimates of negative predictive value were below 99.5% for both risk stratification thresholds in patients with a prior history of ischemic heart disease, diabetes mellitus, stroke, heart failure and renal impairment, although the upper bound of the 95% CIs crossed the prespecified safety margin of 99.5% (Figure 2). In those with available electronic electrocardiograms and evidence of myocardial ischemia, the negative predictive value was 99.6% (95% CI, 99.3%–99.9%) in those with cardiac troponin concentrations less than 5 ng/L and 99.7% (95% CI, 99.2%–100.0%) in those below 2 ng/L.

**Figure 3.**
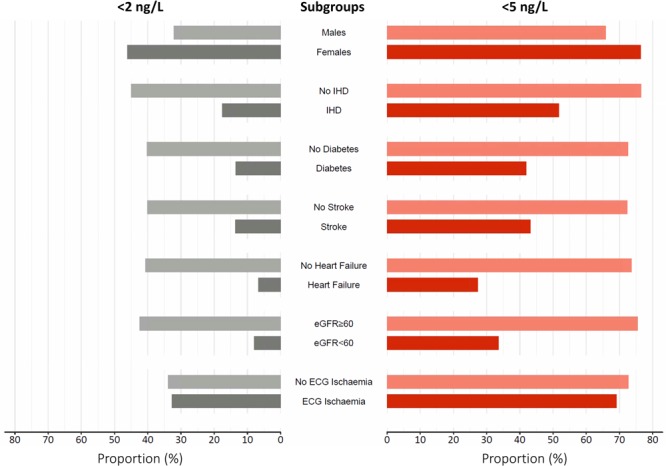
**Proportion of patients identified as low risk at the <2 ng/L and <5 ng/L risk stratification thresholds by subgroups.** Proportion of patients in each subgroup with cardiac troponin concentrations below 2 ng/L (gray) or 5 ng/L (red) at presentation.

The proportion of patients with cardiac troponin concentrations below both thresholds differed widely in these subgroups, but in every subgroup with prior cardiovascular disease, at least twice as many patients were identified as low risk using a risk stratification threshold of 5 ng/L compared with 2 ng/L (Figure 3). Invasive cardiac procedures and changes to preventative cardiac medications were rarely undertaken or initiated following emergency department assessment (Table VI in the online-only Data Supplement). Cardiac catheterization occurred in fewer than 1 in 100 patients below either threshold and new antiplatelet therapy was commenced in fewer than 1 in 25.

### Secondary Safety Outcomes at 12 months

**Figure 4.**
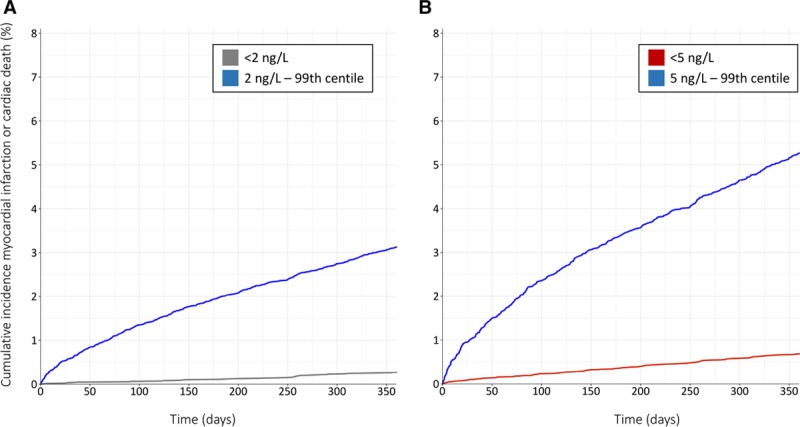
**Cumulative incidence of myocardial infarction or cardiac death at 12 months.** Plots stratified by cardiac troponin concentration at presentation: (**A**) below 2 ng/L (gray) and between 2 ng/L and 99th centile (blue); (**B**) below 5 ng/L (red) and between 5 ng/L and 99th centile (blue).

Subsequent myocardial infarction or cardiac death following discharge from hospital occurred in 2.0% (667 of 32 837) of patients at 12 months. Event rates were similar between patients with cardiac troponin concentrations at presentation below 2 ng/L and 5 ng/L (35 of 12 716 [0.3%] vs 161 of 23 260 [0.7%], respectively), and were lower than those with cardiac troponin concentrations of 5 ng/L to the 99th centile at presentation (506 of 9577 [5.3%]; Table 2 and Figure 4). Lower cardiac troponin concentrations were associated with fewer subsequent events at 12 months; patients with concentrations <2 ng/L had a lower event rate than those with concentrations between these thresholds (126 of 10 544 [1.2%], Figure II in the online-only Data Supplement). When accounting for substantial differences in age and sex between these groups, the risk of subsequent myocardial infarction or cardiac death at 12 months was 80% lower in those below 2 ng/L (adjusted odds ratio, 0.20 [95% CI, 0.14–0.29]), and 77% lower in those less than 5 ng/L (adjusted odds ratio, 0.23 [95% CI, 0.19–0.28]), compared with patients with troponin concentrations between 5 ng/L and the 99th centile. At both 30 days and 1 year, adjusted risk estimates of myocardial infarction and cardiac death were similar for those with cardiac troponin concentrations <2 ng/L and <5 ng/L, and for those patients with concentrations between these thresholds (adjusted odds ratio, 0.30 [95% CI, 0.24–0.36], Table VII in the online-only Data Supplement).

### Diagnostic Performance of Risk Stratification Thresholds for Different High-Sensitivity Assays

In our substudy, 1185 patients presenting more than 2 hours from symptom onset were evaluated using the Siemens Atellica cardiac troponin I assay, and 1042 patients evaluated using the Roche Elecsys troponin T assay (Table VIII in the online-only Data Supplement). Using the Siemens assay, 55% and 15% of patients had a cardiac troponin I concentration <5 ng/L and <2 ng/L at presentation with a negative predictive value of 99.3% (95% CI, 98.5%–99.8%) and 99.2% (95% CI, 97.4%–99.9%), respectively. For the cardiac troponin T assay, 46% and 24% of patients were <5 ng/L and <3 ng/L at presentation, with a negative predictive value of 99.1% (95% CI, 98.0%–99.7%) and 99.4% (95% CI, 98.2%–99.9%) respectively (Table IX in the online-only Data Supplement).

## Discussion

In this prespecified secondary analysis from the High-STEACS trial, we have evaluated the use of risk stratification thresholds for high-sensitivity cardiac troponin I in 32 837 consecutive patients with suspected acute coronary syndrome. We report several important findings for clinicians managing patients with this common presentation. First, in patients with at least 2 hours of symptoms prior to testing, a cardiac troponin concentration below 5 ng/L identifies a group at very low risk of immediate or future cardiac events, with a negative predictive value greater than 99.5%. Second, this performance is maintained regardless of age, sex, and the presence of myocardial ischemia on the electrocardiogram. Third, using a risk stratification threshold of 5 ng/L identifies twice as many patients as low risk at presentation when compared with the limit of detection. Fourth, the negative predictive value of applying a risk stratification threshold of 5 ng/L is consistent across high-sensitivity cardiac troponin I and T assays. Fifth, patients with cardiac troponin concentrations above the risk stratification threshold of 5 ng/L, but below the diagnostic threshold, represent a high-risk group with a 7-fold greater risk of subsequent myocardial infarction or cardiac death over 12 months compared with those below either risk stratification threshold. Taken together, we suggest the use of separate risk stratification and diagnostic thresholds for cardiac troponin, will substantially improve our ability to identify patients at risk compared with the binary approach used in practice today.

High-STEACS is the largest clinical trial to evaluate consecutive patients with suspected acute coronary syndrome reported to date.^19^ This analysis of 32 837 patients is larger than the combined number of patients from 30 observational cohort studies, who were included in 2 recent major retrospective meta-analyses of risk stratification using high-sensitivity cardiac troponin I and T.^8,9^ The negative predictive value of the risk stratification threshold of 5 ng/L for myocardial infarction or cardiac death at 30 days was found to be 99.5% (95% CI, 99.3%–99.6%) across 19 of these cohorts using cardiac troponin I,^8^ which is similar to the 99.8% (95% CI, 99.7%–99.8%) observed here, and was 99.3% (95% CI, 97.3%–99.8%) in 11 cohorts using cardiac troponin T.^9^ Taken together these findings suggest that a single risk stratification threshold could be safely applied for both high-sensitivity cardiac troponin I and T assays.

The American Heart Association/American College of Cardiology and European Society of Cardiology guidelines for the management of acute coronary syndromes recommend the diagnostic threshold for myocardial infarction at the 99th centile as an appropriate limit for exclusion in patients except in early presenters.^3,30^ Alternative approaches have been suggested, such as those described in the recent COMPASS-MI study, which uses a range of thresholds in combination with serial testing and change between two cardiac troponin measures to estimate the negative and positive predictive value for individual patients.^5^ As demonstrated in our prior work, the gain in effectiveness from increasing the threshold above 5 ng/L is small, and the negative predictive value for our safety outcome is lower than 99.5% at higher concentrations.^19^ Similarly, the 0/1 hour pathway recommended by the European Society of Cardiology uses multiple thresholds, but not the 99th centile to rule in and rule out myocardial infarction, at presentation or at 1 hour.^30–32^

These varied approaches acknowledge that patients without myocardial injury at presentation are at risk of cardiovascular events; in the present study more than 1 in 20 patients with cardiac troponin measures between the risk stratification and diagnostic thresholds experienced a subsequent myocardial infarction or cardiac death within 12 months of presentation. Troponin is a continuous marker of cardiovascular risk and low concentrations can be used to estimate long-term cardiovascular risk.^33–35^ This can be informative for clinical decision making, but results need to be interpreted in the context of the individual patient, and these thresholds have not been optimized for this purpose. However, what is clear is that those with intermediate troponin concentrations are at higher risk of future events, and the use of the 99th centile alone does not appear to be an appropriate threshold to risk stratify patients with suspected acute coronary syndrome.

There are a number of strengths to our study. The trial design avoided selection bias through the inclusion of consecutive patients ensuring our analysis population included both low- and high-risk individuals, an equal proportion of men and women, patients who presented outside routine hours, and those who were unlikely to survive. Enrollment was across 10 hospitals in Scotland including both secondary and tertiary care centers. Despite differences in the prevalence of the primary outcome between sites, the proportion of patients identified as low risk and the safety of risk stratification with cardiac troponin was consistent across sites. Within our substudy, we have further explored the generalizability of our findings, demonstrating equivalent diagnostic performance of the same risk stratification threshold for other high-sensitivity cardiac troponin I and T assays. By using robust and established regional and national registries we ensured follow-up was complete in all patients who remained resident in Scotland through linkage of electronic health-care records.^36,37^ Finally, all primary or secondary outcome events were adjudicated in accordance with the Universal Definition of Myocardial Infarction.

There are approximately 20 million presentations with suspected acute coronary syndrome to the emergency departments in the United States and Europe every year.^38^ The adoption of a safe and effective approach to rule out of myocardial infarction would have a considerable impact on healthcare provision. Using an optimized risk stratification threshold of 5 ng/L compared with the limit of detection (<2 ng/L) identifies twice as many low-risk patients. This is particularly relevant in older patients with established cardiovascular disease, where the clinical assessment of pretest probability is more challenging. The optimized risk stratification threshold maintains an excellent safety profile across all age groups and identifies 4 times as many patients >65 years old as low risk. It is well recognized that cardiac troponin concentrations increase with age^39^ where they reflect the presence and control of traditional cardiovascular risk factors, such as hypertension^40^ and hypercholesterolemia,^25^ the burden of coronary artery disease,^26,27^ vulnerable plaque,^41^ and left ventricular hypertrophy or myocardial fibrosis.^42,43^ This property of cardiac troponin as a dynamic barometer of heart health^44^ provides the pathophysiological basis to explain its powerful role in the risk stratification of patients with suspected acute coronary syndrome.^39^

Although the safety profile of both the 5 ng/L and 2 ng/L thresholds appear excellent, prospective trials in which patients are assessed and clinical decisions are guided using this approach are needed to ensure that the very low event rates observed here are not a consequence of hospital admission for further investigation and treatment. In our present analysis, we confirm our previous findings in patients who present within 2 hours of symptoms onset, and suggest that serial testing is required in early presenters to maintain the very high negative predictive value of this approach in all patient groups (Table III in the online-only Data Supplement).^6^ In those presenting more than 2 hours from symptom onset, we further explored the performance of risk stratification thresholds across subgroups. Despite our large sample size, it is possible we were underpowered to evaluate safety in smaller subgroups, such as those with a prior history of ischemic heart disease, diabetes mellitus, stroke, heart failure and renal impairment. In these subgroups, the central estimate, but not the upper bound of the CI for the negative predictive value, was below 99.5% for both risk stratification thresholds. There was evidence of heterogeneity between those with and without prior ischemic heart disease. However, even in those with established risk factors or cardiovascular conditions, all estimates of negative predictive value encompassed our prespecified safety margin of 99.5%. The safety and effectiveness of introducing risk-stratification thresholds into clinical practice is currently being addressed in the HiSTORIC trial (High-Sensitivity Cardiac Troponin on Presentation to Rule Out Myocardial Infarction; https://www.clinicaltrials.gov. Unique identifier: NCT03005158), and in the LoDED study (Limit of Detection of Troponin and ECG Discharge; ISRCTN 86184521).^45^

There are some study limitations relevant to this analysis. We were unable to report use of noninvasive diagnostic testing in our study population, and electrocardiograms were only available for a proportion of patients. However, our analysis shows that the negative predictive value of the optimized risk stratification threshold and 2 ng/L was similar in the presence or absence of myocardial ischemia. In the absence of ST-segment elevation, other abnormalities on the electrocardiogram appear to be less important in patients who have very low cardiac troponin concentrations. This analysis evaluates the risk stratification threshold of a single troponin assay, but we have provided evidence in our substudy of the consistency of this approach for other high-sensitivity cardiac troponin I and T assays. Recent reports also support the validity of this approach across differing high-sensitivity cardiac troponin I and T assays.^9,12,22,46^ The assay’s precision and analytical variation^16,17^ at the risk stratification threshold is likely to influence the clinical utility of using very low cardiac troponin concentrations, and we have not evaluated assay performance or the implications of misclassification here. Although the trial was conducted across 10 different hospitals in Scotland, all are part of a single healthcare system, and additional studies would be helpful in countries where less selective cardiovascular testing is performed.^47^ However, we have previously observed similar safety and effectiveness in a meta-analysis of 19 cohorts across 9 countries.^8^

In conclusion, the use of a risk stratification threshold for high-sensitivity cardiac troponin I in the evaluation of patients with suspected acute coronary syndrome presenting at least 2 hours from symptom onset identifies the majority of patients at low risk of immediate and future cardiovascular events. The use of an optimized risk stratification threshold of 5 ng/L compared with 2 ng/L, classifies twice as many patients as low risk. Although the proportion identified as low risk is reduced in older patients, the safety of this approach is maintained across patients irrespective of age or sex. The adoption of risk stratification thresholds in clinical practice has potential to improve both the effectiveness and safety of the evaluation of patients with suspected acute coronary syndrome with major benefits for patients and healthcare providers.

## Acknowledgments

The authors thank researchers from the Emergency Medicine Research Group of Edinburgh and Edinburgh Clinical Trials Unit for support during the conduct of this trial.

## Sources of Funding

This trial was funded by the British Heart Foundation (SP/12/10/29922) with support from a Research Excellence Award (RE/18/5/34216). CJW was supported by NHS Lothian through the Edinburgh Clinical Trials Unit. Abbott Laboratories provided cardiac troponin assay reagents, calibrators, and controls without charge. AA is supported by a Clinical Lectureship from the Chief Scientist Office (PCL/18/05). AB, ASVS, DEN, and NLM are supported by the British Heart Foundation through the award of a Scholarship (SS/CH/09/002/26360), an Intermediate Clinical Research Fellowship (FS/19/17/34172), Chair (CH/09/002) and the Butler Senior Clinical Research Fellowship (FS/16/14/32023), respectively. DEN is the recipient of a Wellcome Trust Senior Investigator Award (WT103782AIA).

## Disclosures

NLM has acted as a consultant for Abbott Diagnostics, Siemens Healthineers, and LumiraDx, and the University of Edinburgh has received research grants from Abbott Diagnostics and Siemens Healthineers. The other authors report no conflicts.

## Supplementary Material
