# Supplementary material for: High-Sensitivity Troponin and the Application of Risk Stratification Thresholds in Patients With Suspected Acute Coronary Syndrome
Source: Circulation. 2019 Sep 1;140(19):1557–68. doi: 10.1161/CIRCULATIONAHA.119.042866 (PMC6831036; doi:10.1161/CIRCULATIONAHA.119.042866)

SUPPLEMENTAL MATERIAL

High-sensitivity troponin and the application of risk stratification thresholds in patients with suspected acute coronary syndrome

Bularga *- High-sensitivity troponin for risk stratification*

Anda Bularga MD,[1] Kuan Ken Lee MD,[1] Stacey Stewart MSc,[1] Amy V. Ferry MSc,[1] Andrew R. Chapman MD PhD,[1] Lucy Marshall MSc,[1] Fiona E. Strachan PhD,[1] Anne Cruickshank MD,[2] Donogh Maguire MD PhD,[3] Colin Berry MD PhD,[4] Iain Findlay MD,[5] Anoop S.V. Shah MD PhD,[1,6] David E. Newby MD PhD,[1] Nicholas L. Mills MD PhD,[1,6*] and Atul Anand MD PhD[1*]; on behalf of the High-STEACS Investigators[†]

[1] BHF Centre for Cardiovascular Science, University of Edinburgh, Edinburgh, UK.
[2] Department of Biochemistry, Queen Elizabeth University Hospital, Glasgow, UK
[3] Emergency Medicine Department, Glasgow Royal Infirmary, Glasgow, UK
[4] Institute of Cardiovascular and Medical Sciences, University of Glasgow, Glasgow, UK
[5] Department of Cardiology, Royal Alexandra Hospital, Paisley, UK
[6] Usher Institute of Population Health Sciences and Informatics, University of Edinburgh, Edinburgh, UK.

[*] Contributed equally
[†] Listed at the end of the manuscript

Appendix: High-STEACS Investigators

Chief Investigator: Prof Nicholas L Mills.

Trial managers: Dr Fiona E Strachan and Mr Christopher Tuck.

Trial research team: Dr Anoop SV Shah, Dr Fiona E Strachan, Dr Atul Anand, Dr Anda Bularga, Ms Amy V Ferry, Dr Kuan Ken Lee, Dr Andrew R Chapman, Mr Dennis Sandeman, Dr Philip D Adamson, Dr Catherine L Stables, Dr Catalina A Vallejo, Dr Athanasios Tsanasis, Ms Lucy Marshall, Ms Stacey D Stewart, Dr Takeshi Fujisawa, Ms Mischa Hautvast, Ms Jean McPherson and Ms Lynn McKinlay.

Grant applicants: Prof Nicholas L Mills (Principal Applicant), Prof David E Newby, Prof Keith AA Fox, Prof Colin Berry, Dr Simon Walker, and Dr Christopher J Weir.

Trial steering committee: Prof Ian Ford (chair, independent), Prof Nicholas L Mills, Prof David E Newby, Prof Alasdair Gray, Prof Keith AA Fox, Prof Colin Berry, Dr Simon Walker, Prof Paul O Collinson, Prof Fred S Apple, Mr Alan Reid, Dr Anne Cruikshank, Dr Iain Findlay, Dr Shannon Amoils (independent), Dr David A McAllister, Dr Donogh Maguire, Ms Jennifer Stevens (independent), Prof John Norrie (independent), and Prof Christopher Weir.

Adjudication panel: Dr Anoop SV Shah, Dr Atul Anand, Dr Andrew R Chapman, Dr Kuan Ken Lee, Dr Jack PM Andrews, Philip D Adamson, Dr Alastair Moss, Dr Mohamed S Anwar, Dr John Hung, Prof Nicholas L Mills.

Biochemistry sub-group committee: Dr Simon Walker, Dr Jonathan Malo, Mr Alan Reid, Dr Anne Cruikshank, Prof Paul O Collinson.

Data monitoring committee: Prof Colin M Fischbacher, Dr Bernard L Croal, Prof Stephen J Leslie.

Edinburgh Clinical Trials Unit: Ms Catriona Keerie, Mr Richard A Parker, Mr Allan Walker, Mr Ronnie Harkess, Mr Christopher Tuck, Mr Tony Wackett, Prof Christopher Weir.

NHS Greater Glasgow & Clyde Safe Haven: Dr Roma Armstrong, Ms Marion Flood, Ms Laura Stirling, Ms Claire MacDonald, Mr Imran Sadat, Mr Frank Finlay.

NHS Lothian Research Governance, eHealth and Safe Haven: Dr Heather Charles, Ms Pamela Linksted, Mr Stephen Young, Mr Bill Alexander, Mr Chris Duncan.

Appendix: Algorithmic interpretation of ECG ischaemia

As described in the methods, a consensus panel determined a final list of algorithmic codes consistent with clinically relevant myocardial ischaemia on example case review. These 119 codes are reproduced here in the format generated by the MUSE (GE Healthcare) system:

Abnrm T, probable ischemia, anterolateral lds;
Repol abnrm, prob ischemia, anterolateral lds;
Repol abnrm, global ischemia, diffuse leads;
LVH w/ repol abnormalities, possible ischemia;
Abnormal T, probable ischemia, inferior leads;
Abnormal T, probable ischemia, lateral leads;
Tall T, consider metabolic/ischemic abnrm;
Abnormal T, consider ischemia, inferior leads;
Repol abnrm suggests ischemia, inferior leads;
ABNORMAL T, PROBABLE ISCHEMIA, ANT-LAT LEADS;
Repol abnrm, probable ischemia, lateral leads;
Repol abnrm suggests ischemia, anterior leads;
Repol abnrm, probable ischemia, anterior lds;
Repol abnrm, probable ischemia, inferior lds;
Repol abnrm, prob ischemia, inferolateral lds;
ABNORMAL T, CONSIDER ISCHEMIA, ANT-LAT LEADS;
ABNORMAL T, CONSIDER ISCHEMIA, LATERAL LEADS;
REPOL ABNRM SUGGESTS ISCHEMIA, LATERAL LEADS;
ABNORMAL T, PROBABLE ISCHEMIA, INFERIOR LEADS;
ABNORMAL T, CONSIDER ISCHEMIA, ANTERIOR LEADS;
REPOL ABNRM SUGGESTS ISCHEMIA, DIFFUSE LEADS;
ABNORMAL T, CONSIDER ISCHEMIA, DIFFUSE LEADS;
Abnormal lateral Q waves;
Abnormal inferior Q waves;
Anteroseptal infarct, old;
Anterior infarct, old;
Inferior infarct, old;
Anterior infarct, age indeterminate;
Inferior infarct, age indeterminate;
Anterolateral infarct, age indeterminate;
PROBABLE INFERIOR INFARCT, OLD;
Probable anteroseptal infarct, recent;
Probable anterior infarct, age indeterminate;
Probable posterior infarct, recent;
Probable inferior infarct, acute;
INFERIOR INFARCT, AGE INDETERMINATE;
ANTERIOR INFARCT, AGE INDETERMINATE;
Lateral infarct, acute;
Anteroseptal infarct, age indeterminate;

Inferior infarct, acute;
Inferolateral infarct, age indeterminate;
Posterior infarct, old;
Probable anterolateral infarct, recent;
Anterior infarct, acute;
Probable anterolateral infarct, age indeterm;
Probable anterolateral infarct, acute;
Anterior infarct, recent;
Inferior infarct, recent;
Inferior infarct, acute (RCA);
Inferolateral infarct, old;
Consider anterolateral infarct;
Probable anteroseptal infarct, acute;
Probable posterior infarct, acute;
Inferoposterior infarct, recent;
Lateral infarct, acute (LAD);
Inferoposterior infarct, old;
Inferoposterior infarct, acute;
Anteroseptal infarct, possibly acute;
Posterior infarct, recent;
Lateral infarct, old;
Anterolateral infarct, acute;
Probable lateral infarct, age indeterminate;
Probable inferior infarct, recent;
Extensive anterior infarct, old;
Lateral infarct, recent;
Anterolateral infarct, recent;
Anterolateral infarct, acute (LAD);
CONSIDER POSTERIOR INFARCT;
Inferoposterior infarct, age indeterminate;
Anteroseptal infarct, acute;
ANTERIOR INFARCT, OLD;
Inferolateral infarct, acute;
Lateral infarct, age indeterminate;
Extensive anterior infarct, acute;
Consider posterior infarct;
Extensive anterior infarct, acute (LAD);
Probable anterior infarct, acute;
Inferolateral infarct, acute (RCA);
Inferoposterior infarct, acute (RCA);
Probable lateral infarct, recent;
Inferior infarct, acute (LCx);
Inferior infarct, possibly acute;
Probable anterior infarct, recent;

Inferoposterior infarct, acute (LCx);
Consider inferoposterior infarct;
Inferolateral infarct, recent;
Anterolateral infarct, possibly acute;
Lateral infarct, possibly acute;
Extensive anterior infarct, age indeterminate;
INFERIOR INFARCT, OLD;
Inferolateral infarct, acute (LCx);
PROBABLE INFERIOR INFARCT, AGE INDETERMINATE;
ANTEROLATERAL INFARCT, RECENT;
CONSIDER INFERIOR INFARCT;
LATERAL INFARCT, OLD;
PROBABLE INFEROLATERAL INFARCT, AGE INDETERM;
Minimal ST elevation, diffuse leads;
ST depr, consider ischemia, anterolateral lds;
ST depression, consider ischemia, diffuse lds;
ST depr, consider ischemia, anterior leads;
ST DEPRESSION, CONSIDER ISCHEMIA, DIFFUSE LDS;
Minimal ST depression, lateral leads;
Borderline ST depression, anterolateral leads;
ST depression, probably rate related;
Minimal ST depression, anterolateral leads;
Minimal ST depression, inferior leads;
Minimal ST depression, diffuse leads;
Borderline ST depression, lateral leads;
ST depression V1-V3, suggest recording posterior leads;
Borderline ST depression, diffuse leads;
Nonspecific ST depression;
Minimal ST depression, anterior leads;
MINIMAL ST DEPRESSION, LATERAL LEADS;
Borderline ST depression, inferior leads;
Borderline ST depression, anterior leads;
Nonspecific ST depression, anterior leads;
Nonspecific ST depression, anterolateral lds;
MINIMAL ST DEPRESSION, ANTEROLATERAL LEADS;
BORDERLINE ST DEPRESSION, LATERAL LEADS;

Supplementary Table 1: Baseline characteristics of participants stratified by presentation high-sensitivity cardiac troponin I (hs-cTnI) for all patients and those <5 ng/L.

		Presentation hs-cTnI		
	**All**	**<2 ng/L**	**2 ng/L – 4 ng/L**	**<5 ng/L**
n	32837	12716	10544	23260
Age	58.4 (17.1)	47.8 (13.9)	60.6 (14.8)	53.6 (15.7)
Males	17478 (53)	5620 (44)	5899 (56)	11519 (50)
**Presenting Complaint**				
Chest pain	24085 (73)	9793 (77)	8037 (76)	17830 (77)
Dyspnoea	1001 (3)	169 (1)	229 (2)	398 (2)
Palpitation	825 (3)	269 (2)	271 (3)	540 (2)
Syncope	1162 (4)	216 (2)	358 (3)	574 (3)
Other	1197 (4)	306 (2)	416 (4)	722 (3)
**Past Medical History**				
Ischaemic heart disease	7467 (23)	1309 (10)	2554 (24)	3863 (17)
Myocardial infarction	2537 (8)	432 (3)	855 (8)	1287 (6)
Stroke or TIA	1700 (5)	231 (2)	504 (5)	735 (3)
Percutaneous coronary intervention	2416 (7)	461 (4)	890 (8)	1351 (6)
Coronary artery bypass grafting	477 (2)	58 (1)	10395 (98.6)	207 (1)
Diabetes mellitus	1867 (6)	253 (2)	10015 (95.0)	782 (3)
Heart failure	1956 (6)	130 (1)	10139 (96.2)	535 (2)
**Medications**				
Aspirin	8277 (25)	1654 (13)	2965 (28)	4619 (20)
Clopidogrel	2555 (8)	437 (3)	870 (8)	1307 (6)
Ticagrelor	225 (1)	43 (0.3)	86 (1)	129 (1)
Oral anticoagulant	1951 (6)	219 (2)	534 (5)	753 (3)
ACE inhibitor or ARB	9799 (30)	1969 (16)	3501 (33)	5470 (24)
Beta-blocker	8398 (26)	1943 (15)	2920 (28)	4863 (21)
Statin	12264 (37)	2594 (20)	4408 (42)	7002 (30)
Loop diuretics	3420 (10)	356 (3)	820 (8)	1176 (5)
**Laboratory Results**				
Presentation hs-cTnI	2.4 [1.0, 5.7]	1.0 [1.0, 1.1]	3.0 [2.0, 3.7]	1.6 [1.0, 2.8]
Peak hs-cTnI	2.7 [1.0, 6.0]	1.0 [1.0, 1.3]	3.0 [2.1, 4.0]	1.8 [1.0, 3.0]
Serial hs-cTnI test	13554 (41)	4552 (36)	4402 (42)	8954 (39)
GFR, ml/min/1.73m^2	88 (24)	96 (19)	88 (22)	92 (21)

Data are number of patients (%), mean (SD) or median [IQR]; TIA = Transient Ischemic Attack, ACE = Angiotensin Converting Enzyme; ARB = Angiotensin Receptor Blocker; GFR = Glomerular Filtration Rate.

Supplementary Table 2: Performance of the risk stratification threshold by site. Shown are the number of primary outcome events at 30 days with the negative predictive value (NPV) at <2 ng/L and <5 ng/L by each site in the High-STEACS trial. CI = confidence interval.

Site	All Patients		<2 ng/L			<5 ng/L		
	**n**	**Events (%)**	**n (%)**	**Events (%)**	**NPV (95% CI)**	**n (%)**	**Events (%)**	**NPV (95% CI)**
1	8,492	162 (1.9)	2,798 (33)	4 (0.1)	99.8 (99.7–100.0)	5,976 (70)	14 (0.2)	99.8 (99.6–99.9)
2	2,560	21 (0.8)	778 (30)	0 (0.0)	99.9 (99.8–100.0)	1,698 (66)	0 (0.0)	100.0 (99.9–100.0)
3	3,383	55 (1.6)	1,277 (38)	1 (0.1)	99.8 (99.7–100.0)	2,525 (75)	3 (0.1)	99.9 (99.7–100.0)
4	2,971	36 (1.2)	1,307 (44)	1 (0.1)	99.9 (99.7–100.0)	2,121 (71)	9 (0.4)	99.6 (99.3–99.8)
5	2,858	36 (1.3)	1,233 (43)	2 (0.2)	99.8 (99.5–100.0)	2,007 (70)	6 (0.3)	99.7 (99.4–99.9)
6	717	8 (1.1)	301 (42)	0 (0.0)	99.8 (99.4–100.0)	524 (73)	0 (0.0)	99.9 (99.6–100.0)
7	1,318	20 (1.5)	487 (37)	1 (0.2)	99.7 (99.2–100.0)	907 (69)	3 (0.3)	99.6 (99.2–99.9)
8	2,951	61 (2.1)	1,205 (41)	2 (0.2)	99.8 (99.5–100.0)	2,050 (69)	6 (0.3)	99.7 (99.4–99.9)
9	3,458	56 (1.6)	1,418 (41)	4 (0.3)	99.7 (99.4–99.9)	2,424 (70)	6 (0.2)	99.7 (99.5–99.9)
10	4,129	62 (1.5)	1,912 (46)	0 (0.0)	100.0 (99.9–100.0)	3,028 (73)	8 (0.3)	99.7 (99.5–99.9)
**Total**	**32,837**	**517 (1.6)**	**12,716 (39)**	**15 (0.1)**	**99.9 (99.8–99.9)**	**23,260 (71)**	**55 (0.2)**	**99.8 (99.7–99.8)**

Supplementary Table 3: Negative predictive value (NPV) in all patients and selected subgroups restricted to those with ≤2 hours of symptoms at presentation (n=6,469). IHD = ischaemic heart disease; eGFR = estimated glomerular filtration rate; 95% CI = 95% confidence interval; TN = true negative; FN = false negative.

Group	Threshold	n	TN	FN	NPV (95% CI)
All	<5 ng/L	6,469	43	4,197	99.0 (98.7-99.3)
	<2 ng/L	6,469	9	2,230	99.6 (99.3-99.8)
Males	<5 ng/L	3,824	24	2,257	98.9 (98.5-99.3)
	<2 ng/L	3,824	4	1,053	99.6 (99.2-99.9)
Females	<5 ng/L	2,645	5	1,177	99.5 (99.1-99.9)
	<2 ng/L	2,645	15	8,263	99.8 (99.7-99.9)
IHD	<5 ng/L	1,572	22	737	97.0 (95.8-98.2)
	<2 ng/L	1,572	5	245	97.8 (96.0-99.4)
Diabetes	<5 ng/L	424	6	148	95.8 (92.6-98.6)
	<2 ng/L	424	1	44	96.7 (91.6-100.0)
Stroke	<5 ng/L	355	2	150	98.4 (96.4-99.9)
	<2 ng/L	355	1	49	97.1 (92.4-100.0)
Heart failure	<5 ng/L	465	3	112	97.0 (93.9-99.6)
	<2 ng/L	465	0	21	97.7 (91.4-100.0)
eGFR<60	<5 ng/L	1,082	7	279	97.4 (95.5-99.1)
	<2 ng/L	1,082	1	47	96.9 (92.1-100.0)
ECG ischaemia	<5 ng/L	273	1	189	99.2 (98.0-100.0)
	<2 ng/L	273	0	66	99.3 (97.1-100.0)

Supplementary Table 4: Diagnostic performance table for all High-STEACS patients excluding those with ST-segment myocardial infarction for the composite primary outcome at 30 days (n=47,101).

	**<2 ng/L (n=14,955)**	**<5 ng/L (n=27,500)**
**Primary outcome at 30 days**		

	Present	Absent
<2 ng/L	24	14,931
≥2 ng/L	4,289	27,857

	Present	Absent
<5 ng/L	98	27,402
≥5 ng/L	4,215	15,386

	**<2 ng/L (n=14,955)**	**<5 ng/L (n=27,500)**
**Sensitivity**	**99.4 (99.2-99.6)**	**97.7 (97.3-98.1)**
Specificity	34.9 (34.4-35.3)	64.0 (63.6-64.5)
**Negative predictive value**	**99.8 (99.8-99.9)**	**99.6 (99.6-99.7)**
Positive predictive value	13.3 (13.0-13.7)	21.5 (20.9-22.1)

Supplementary Table 5: Diagnostic performance table of the analysis population for the composite primary outcome at 30 days (n=32,837).

		**<2 ng/L (n=12,716)**			**<5 ng/L (n=23,260)**	
**Primary outcome at 30 days**						
		Present	Absent		Present	Absent
	<2 ng/L	15	12,701	<5 ng/L	55	23,205
	≥2 ng/L	502	19,619	≥5 ng/L	462	9,115
**Sensitivity**		**97.0 (95.4-98.3)**			**89.3 (86.5-91.8)**	
Specificity		39.3 (38.8-39.8)			71.8 (71.3-72.3)	
**Negative predictive value**		**99.9 (99.8-99.9)**			**99.8 (99.7-99.8)**	
Positive predictive value		2.5 (2.3-2.7)			4.8 (4.4-5.3)	

Supplementary Table 6: Investigations and treatments stratified by presentation cardiac troponin concentation. Data are number of patients (%) unless stated otherwise. IQR = interquartile range; PCI = percutaneous coronary intervention; CABG = coronary artery bypass grafting; ACE = angiotensin converting enzyme; ARB = angiotensin II receptor blocker.

	**All (n=32,837)**	**<2 ng/L (n=12,716)**	**2 ng/L – 99th centile (n=20,121)**	**P-value**	**<5 ng/L (n=23,260)**	**5 ng/L – 99th centile (n=9,577)**	**P-value**
Length of stay, hours (median [IQR])	4.9 [3.2, 21.9]	3.9 [2.9, 12.1]	7.4 [3.5, 27.5]	<0.001	4.0 [3.0, 16.6]	14.8 [3.8, 50.5]	<0.001
Coronary angiography	673 (2)	78 (1)	595 (3)	<0.001	224 (1)	449 (5)	<0.001
PCI	346 (1)	26 (0.2)	320 (2)	<0.001	102 (0.4)	244 (3)	<0.001
PCI or CABG	374 (1)	32 (0.3)	342 (2)	<0.001	113 (1)	261 (3)	<0.001
New anti-platelet	1780 (5)	421 (3)	1359 (7)	<0.001	928 (4)	852 (9)	<0.001
New dual anti-platelet	756 (2)	57 (0.4)	699 (4)	<0.001	237 (1)	519 (5)	<0.001
New statin	984 (3)	265 (2)	719 (4)	<0.001	540 (2)	444 (5)	<0.001
New ACE inhibitor/ARB	765 (2)	134 (1)	631 (3)	<0.001	335 (1)	430 (5)	<0.001
New beta-blocker	1719 (5)	507 (4)	1212 (6)	<0.001	971 (4)	748 (8)	<0.001

Supplementary Table 7: Logistic regression modelling for outcomes at 30 days and 12 months stratified by presentation high-sensitivity cardiac troponin I concentration

	5 ng/L - 99th centile (n=9,577)		<2 ng/L (n=12,716)			2 ng/L – 4 ng/L (n=10,544)			<5 ng/L (n=23,260)		
	Events	OR (Reference)	Events	OR (95% CI)	Adjusted OR	Events	OR (95% CI)	Adjusted OR	Events	OR (95% CI)	Adjusted OR
**30 days**											
Myocardial infarction	59 (0.6%)	1.00	3 (0.0%)	0.06 (0.02-0.17)	0.10 (0.02-0.28)	16 (0.2%)	0.25 (0.14-0.42)	0.27 (0.14-0.46)	19 (0.1%)	0.13 (0.08-0.22)	0.17 (0.10-0.31)
Cardiac death	45 (0.5%)	1.00	1 (0.0%)	0.03 (0.00-0.15)	0.16 (0.01-0.76)	3 (0.0%)	0.06 (0.01-0.16)	0.11 (0.03-0.30)	4 (0.0%)	0.04 (0.01-0.09)	0.10 (0.03-0.26)
MI or cardiac death	99 (1.0%)	1.00	4 (0.0%)	0.05 (0.02-0.13)	0.12 (0.03-0.29)	19 (0.2%)	0.17 (0.10-0.28)	0.22 (0.13-0.35)	23 (0.1%)	0.09 (0.06-0.15)	0.16 (0.09-0.25)
**12 months**											
Myocardial infarction	282 (2.9%)	1.00	25 (0.2%)	0.11 (0.07-0.16)	0.20 (0.13-0.31)	80 (0.8%)	0.25 (0.20-0.32)	0.30 (0.23-0.39)	105 (0.5%)	0.15 (0.12-0.19)	0.23 (0.18-0.30)
Cardiac death	253 (2.6%)	1.00	11 (0.1%)	0.06 (0.03-0.10)	0.19 (0.10-0.34)	49 (0.5%)	0.17 (0.13-0.23)	0.28 (0.20-0.38)	60 (0.3%)	0.10 (0.07-0.13)	0.23 (0.16-0.31)
MI or cardiac death	506 (5.3%)	1.00	35 (0.3%)	0.09 (0.06-0.12)	0.20 (0.14-0.29)	126 (1.2%)	0.22 (0.18-0.26)	0.30 (0.24-0.36)	161 (0.7%)	0.12 (0.10-0.15)	0.23 (0.19-0.28)

Data are number of myocardial infarctions or cardiac deaths excluding index events (%); MI = Myocardial Infarction. Odds Ratios (OR) are derived from logistic regression models comparing the group with presentation hs-cTnI <2 ng/L, 2 ng/L – 4 ng/L or <5 ng/L against the reference group 5 ng/L – 99th centile (95% confidence intervals). Adjusted OR includes age and sex in the logistic regression model.

Supplementary Table 8: Baseline characteristics of the High-STEACS substudy population stratified by assay. Values are mean (SD), median (IQR) or n (%). MI = myocardial infarction; PCI = percutaneous coronary intervention; CABG = coronary artery bypass grafting; ACE = angiotensin converting enzyme; BP = blood pressure.

	**ABBOTT ARCHITECT (I) (n=1,935)**	**SIEMENS ATELLECA (I) (n=1920)**	**ROCHE ELECSYS (T) (n=1859)**
**Baseline characteristics**			
Age	61.5 (14.2)	61.5 (14.2)	61.5 (14.3)
Male (%)	1182 (61.1)	1174 (61.1)	1132 (60.9)
Chest pain (%)	1623 (84.1)	1613 (84.3)	1563 (84.3)
Symptom to arrival time (mins) *	199 (118-464)	199 (118 – 462)	199 (118-463)
**Past medical history**			
Diabetes mellitus (%)	286 (14.8)	281 (14.6)	274 (14.7)
Hypertension (%)	769 (39.7)	763 (39.7)	736 (39.6)
Hyperlipidemia (%)	765 (39.5)	760 (39.6)	730 (39.3)
Family History (%)	927 (47.9)	922 (48.0)	888 (47.8)
Ischemic Heart Disease (%)	582 (30.1)	578 (30.1)	551 (29.6)
Previous MI (%)	460 (23.8)	455 (23.7)	433 (34.4)
Previous PCI (%)	366 (18.9)	363 (18.9)	347 (18.7)
Previous CABG (%)	117 (6.0)	117 (6.1)	110 (5.9)
Previous Heart Failure (%)	66 (3.4)	64 (3.3)	64 (3.4)
Previous Stroke (%)	119 (6.1)	119 (6.2)	115 (6.2)
Smoker (%)	385 (19.9)	385 (20.1)	375 (20.2)
**Medication history**			
Aspirin (%)	655 (33.9)	650 (33.9)	626 (33.7)
Clopidogrel (%)	250 (12.9)	250 (13.0)	235 (12.6)
Beta-blocker (%)	522 (27.0)	518 (27.0)	503 (27.1)
ACE Inhibitor (%)	582 (30.1)	578 (30.1)	556 (29.9)

Statin (%)	824 (42.6)	819 (42.7)	790 (42.5)
Long Acting Nitrate (%)	369 (19.1)	369 (19.2)	349 (18.8)
Calcium Channel Blocker (%)	242 (12.5)	240 (12.5)	229 (12.3)
Warfarin (%)	105 (5.4)	104 (5.4)	104 (5.6)
**Electrocardiogram findings**			
ST depression (%)	112 (5.8)	112 (5.8)	109 (5.9)
ST elevation (%)	58 (3.0)	58 (3.0)	58 (3.1)
T-wave inversion (%)	300 (15.5)	298 (15.5)	289 (15.5)
**Physiological parameters**			
Systolic BP (mmHg)	137 (124-152)	137 (124-152)	137 (124-152)
Diastolic BP (mmHg)	77 (68-88)	79 (70-89)	78 (69-88)
Heart rate (bpm)	75 (64-87)	75 (65-86)	75 (65-86)
Temperature	36.5 (36.1-36.9)	36.5 (36.1-37)	36.5 (36.1-36.9)
Respiratory rate	16 (16-18)	16 (16-18)	16 (16-18)
Oxygen saturations	97 (96-98)	98 (96-99)	97 (96-99)
Creatinine †	74 (67-84)	73 (66-78)	74 (67-82)

* Data missing in 10.4% (202/1,935) † Data missing in 10% (193/1,935).

Supplementary Table 9: Performance of risk stratification thresholds across three high-sensitivity troponin assays in the High-STEACS substudy. NPV = negative predictive value, CI = confidence interval.

	**Total (n)**	**≤99th centile and >2 hours symptoms**	**n (%) <2 ng/L (I) <3 ng/L (T)**	**NPV (95% CI)**	**n (%) <5 ng/L**	**NPV (95%CI)**
**Abbott ARCHITECT (I)**	1,935	1,197	381 (31.8)	99.6% (98.8–99.9)	828 (69.2)	99.8% (99.4–99.9)
**Siemens Atellica (I)**	1,920	1,185	176 (14.9)	99.2% (97.4–99.9)	646 (54.5)	99.3% (98.5–99.8)
**Roche Elecsys (T)**	1,859	1,042	254 (24.4)	99.4% (98.2–99.9)	480 (46.1)	99.1% (98.0–99.7)

Supplementary Figure 1: CONSORT diagram. This flow diagram shows the distribution of our analysis population. For the purpose of this analysis we excluded patients with the ST-segment elevation myocardial infarction (STEMI), those with missing high-sensitivity cardiac troponin I (hs-cTnI) results at presentation, and those with a hs-cTnI concentrations >99th centile and presenting within two hours of symptom onset.

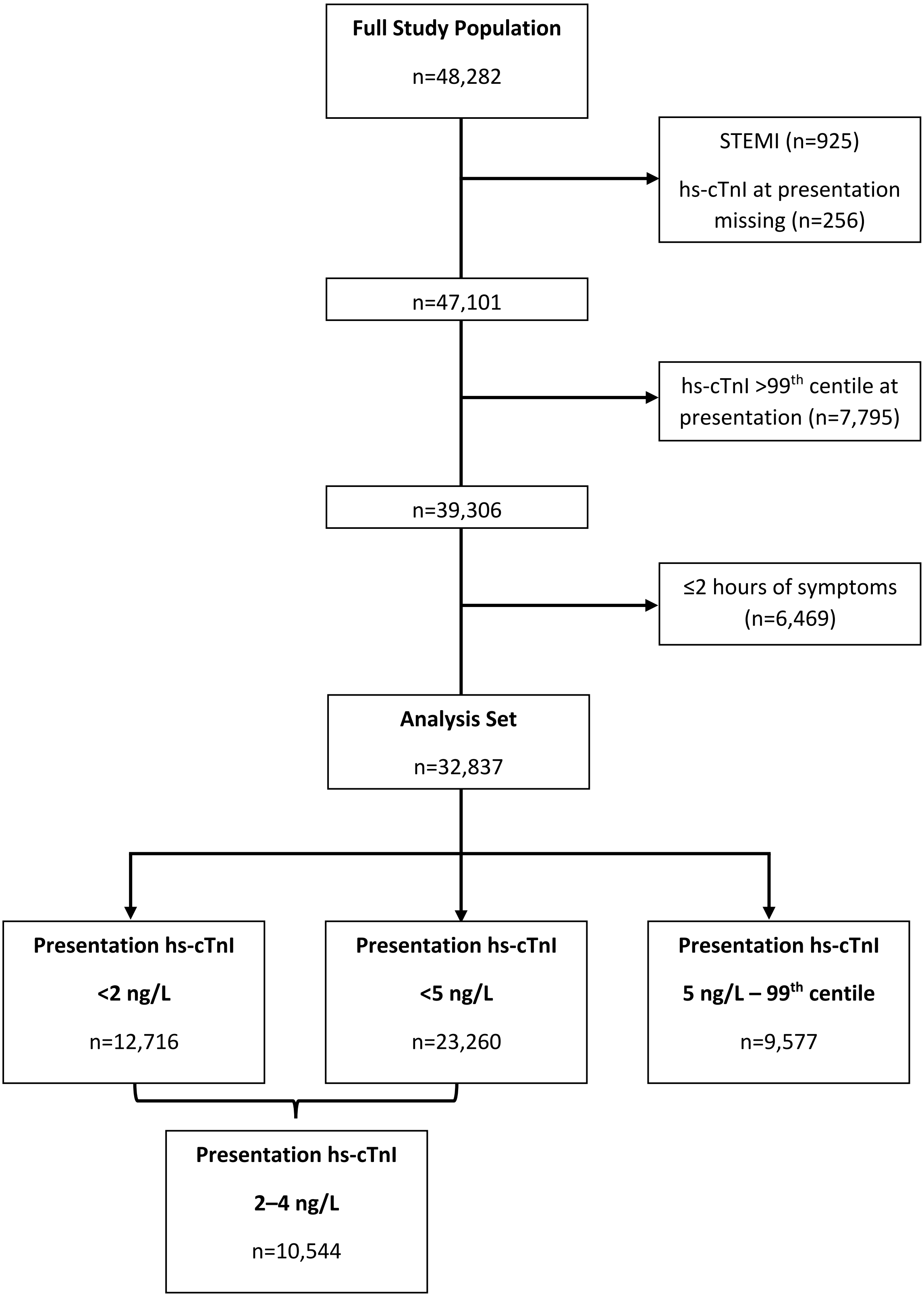

Supplementary Figure 2: Cumulative incidence of myocardial infarction or cardiac death at 12 months. Plots stratified by cardiac troponin concentration at presentation: below 2 ng/L (grey), between 2 ng/L and 4 ng/L (red), and between 5 ng/L and 99th centile (blue).

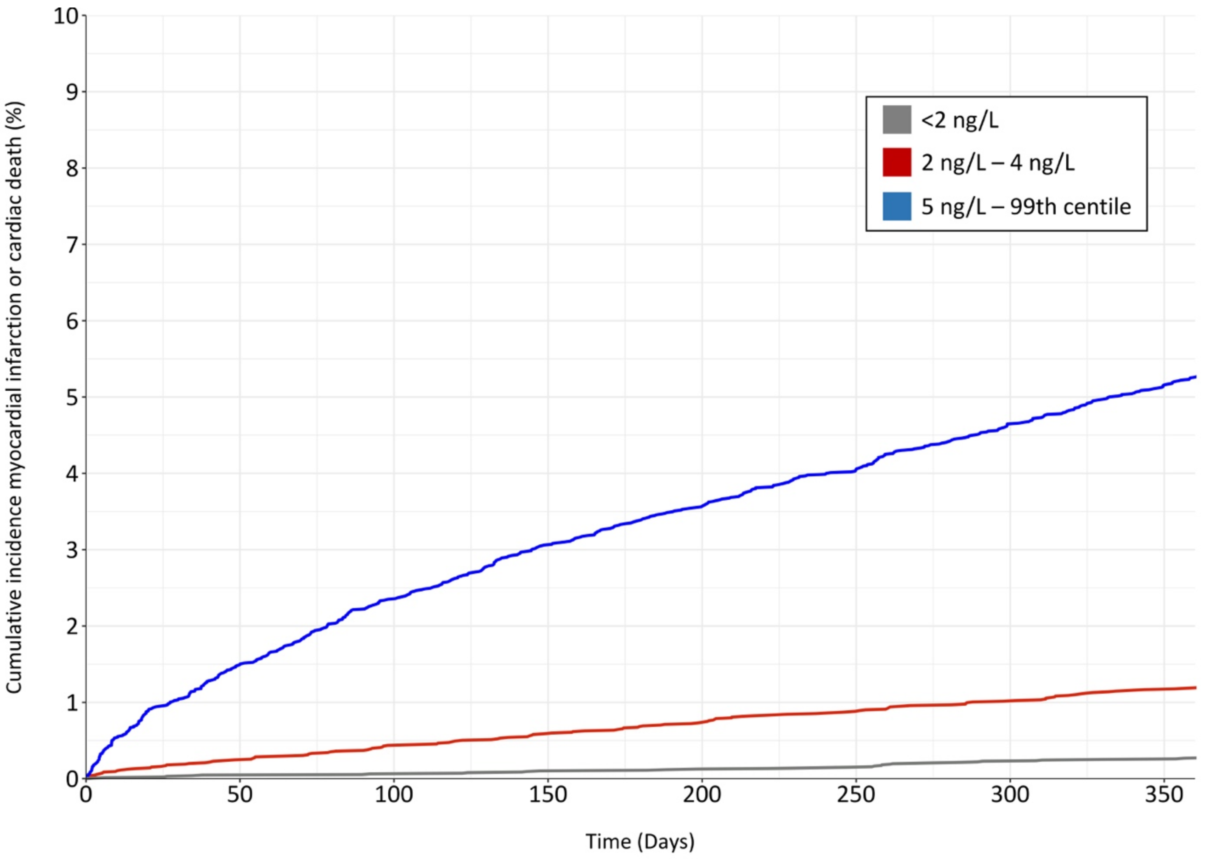

Supplement: Supplementary file 1 [file cir-140-1557-s001.pdf]
